# Maternal Pre-Pregnancy BMI and Gestational Weight Gain Are Associated with Preschool Children’s Neuropsychological Outcomes in the APrON Cohort

**DOI:** 10.3390/children10121849

**Published:** 2023-11-25

**Authors:** Gillian England-Mason, Alida Anderson, Rhonda C. Bell, Fatheema B. Subhan, Catherine J. Field, Nicole Letourneau, Gerald F. Giesbrecht, Deborah Dewey, The APrON Study Team

**Affiliations:** 1 Department of Pediatrics, Cumming School of Medicine, University of Calgary, Calgary, AB T2N 1N4, Canadanicole.letourneau@ucalgary.ca (N.L.); ggiesbre@ucalgary.ca (G.F.G.); 2 Owerko Centre, Alberta Children’s Hospital Research Institute, University of Calgary, Calgary, AB T2N 1N4, Canada; 3 O’Brien Centre for the Bachelor of Health Sciences, Cumming School of Medicine, University of Calgary, Calgary, AB T2N 1N4, Canada; alida.anderson@ucalgary.ca; 4 Department of Agricultural, Food & Nutritional Science, University of Alberta, Edmonton, AB T6G 2R3, Canada; rhonda.bell@ualberta.ca (R.C.B.); catherine.field@ualberta.ca (C.J.F.); 5 Department of Nutrition and Food Science, California State Polytechnic University, Pomona, CA 91768, USA; fsubhan@cpp.edu; 6 Faculty of Nursing, University of Calgary, Calgary, AB T2N 1N4, Canada; 7 Department of Psychiatry, Cumming School of Medicine, University of Calgary, Calgary, AB T2N 1N4, Canada; 8 Department of Community Health Sciences, Cumming School of Medicine, University of Calgary, Calgary, AB T2N 1N4, Canada; 9 Department of Psychology, Faculty of Arts, University of Calgary, Calgary, AB T2N 1N4, Canada; 10 Hotchkiss Brain Institute, University of Calgary, Calgary, AB T2N 1N4, Canada; 11 University of Calgary, Calgary, AB T2N 1N4, Canada; apron@ucalgary.ca; 12 University of Alberta, Edmonton, AB T6G 2R3, Canada

**Keywords:** pre-pregnancy BMI, gestational weight gain, cognitive development, intelligence, language, memory, motor skills, executive function, APrON

## Abstract

This study examined the associations between maternal pre-pregnancy BMI and gestational weight gain (GWG) and children’s neuropsychological outcomes at 3 to 5 years of age. A total of 379 women and their children from the Alberta Pregnancy Outcomes and Nutrition (APrON) study participated. Covariate-adjusted robust regressions examined associations between maternal pre-pregnancy BMI, GWG class, interaction terms, and child outcomes. Each unit increase in maternal BMI was linked to a 0.48-point decrement (95% CI: −0.75 to −0.21) in children’s Full Scale IQ. Higher pre-pregnancy BMI was related to poorer performance on the other intelligence indexes (*B* = −0.35 to −0.47, 95% CIs: −0.75, −0.02) and lower performance on measures of language (*B =* −0.08 to −0.09, 95% CIs: −0.16, −0.02), motor skills (*B* = −0.08 to −0.11, 95% CIs: −0.18, −0.01), and executive function (*B* = −0.09 to −0.16, 95% CIs: −0.26, −0.01). GWG below the recommended range was associated with a 4.04-point decrement (95% CI: 7.89, −0.11) in Full Scale IQ, but better performance on a spatial working memory test (*B* = 0.27, 95% CI: 0.02, 0.52). GWG above the recommended range was associated with lower language (*B =* −0.79, 95% CI: −1.52, −0.06) and memory scores (*B* = −0.93, 95% CI: −1.64, −0.22). Interactions were found between pre-pregnancy BMI and GWG on measures of intelligence and executive function. Maternal pre-pregnancy BMI and GWG are related to children’s performance in various neuropsychological domains and may interact to predict outcomes. Optimizing maternal health and weight prior to conception and during pregnancy may enhance children’s neuropsychological outcomes.

## 1. Introduction

The World Health Organization (WHO) estimates that worldwide, 40% of women are overweight [1]. The 2018 Canadian Community Health Survey (CCHS) reported that 43.6% of women aged 20 to 34 years were overweight or obese [2]. The U.S. National Health and Nutrition Examination Survey (NHANES) found that the rate of obesity among women 20 to 39 years of age was 39.7% [3]. Thus, many women are overweight or obese prior to pregnancy. Gestational weight gain (GWG) also warrants attention as global estimates indicate that 47% of pregnant women have GWG above the Institute of Medicine (IOM) guidelines [4].

During gestation, the fetus is particularly sensitive to intrauterine hormonal and biochemical perturbations, including those associated with higher maternal pre-pregnancy body mass index (BMI) and increased GWG [5,6]. The presence of higher maternal BMI and increased GWG could result in an intrauterine environment that does not support optimal fetal development. This suboptimal environment may be linked to fetal brain development and in turn long-term neuropsychological development. Maladaptive interactions between the maternal–placental–fetal triad produced by these suboptimal conditions could impact children’s overall neurodevelopment, but particularly their cognitive potential [7].

It has been suggested that physiological alterations to the fetus, referred to as fetal programming, are the result of intrauterine conditions associated with pre-pregnancy obesity [8]. While the underlying mechanisms through which pre-pregnancy BMI could be related to neurodevelopment are unknown, it is hypothesized that the inflammation that accompanies obesity may impair complex developmental events within the fetal brain (e.g., neuronal proliferation, migration, synaptogenesis, myelination) and increase oxidative stress, which in turn may contribute to adverse cognitive, behavioural and emotional outcomes in offspring [6,9,10]. Other conditions that could influence brain development are also more common in women with high BMIs, including gestational diabetes, thyroid dysfunction, and nutrient deficiencies (i.e., vitamin D, folate) [6,8]. It has been hypothesized that these conditions may be involved in mechanistic pathways linking maternal pre-pregnancy overweight status or obesity to poorer neurodevelopmental outcomes, including increased risk of conditions including autism spectrum disorder, developmental delay, or attention-deficit/hyperactivity disorder, as well as cognitive, emotional, and behavioural problems [6,8].

The research literature suggests that higher maternal pre-pregnancy BMI is related to lower intelligence and poorer executive function in children [6,10,11]. Studies focusing on maternal overweight and/or obesity have also reported associations with lower performance on measures of language, motor, and socio-emotional skills in preschool-aged children [12], as well as elevated behaviour problems and increased risk for affective disorders [13,14]. However, Huang et al. [15] found that both high and low pre-pregnancy BMI were related to poorer cognitive outcomes, and that children of women with a pre-pregnancy BMI in the normal range had the highest IQ scores. Similar findings were reported by Neggers and colleagues [16]. However, a study conducted by Brion and colleagues [17] reported that maternal pre-pregnancy overweight status was not consistently associated with parent ratings of cognitive development and behavioural problems in children in high- and middle-income cohorts. Maternal GWG may also be related to children’s neuropsychological outcomes. However, the findings to date are inconsistent. Some studies reported that excessive GWG (>2009 IOM Guidelines) is negatively associated with children’s cognitive outcomes [18], whereas others reported positive or null associations [19].

The inconsistencies in the research literature could be due to methodological differences among studies including differences in the neuropsychological assessment measures used and differences in the mean ages and age ranges of the children who participated in the studies. Further, important confounders such as socioeconomic status and maternal mental health, which may be associated with maternal pre-pregnancy BMI and GWG, and child neurodevelopment, were not always considered [20,21]. These limitations support the need for research that investigates the links between maternal pre-pregnancy BMI and child neuropsychological outcomes.

Previous research indicates that higher maternal pre-pregnancy BMI and excessive GWG may interact to predict higher infant birthweight [22], and both low (<2500 g) and high birthweight (>4000 g) are linked to adverse neurodevelopmental outcomes [23]. However, it is currently unknown if maternal BMI and GWG interact to affect children’s neurodevelopment. More research on how maternal weight-related variables are related to children’s neuropsychological outcomes is needed [19]. To address this knowledge gap, this study investigated the links between maternal pre-pregnancy BMI, GWG, their interaction, and children’s intelligence, language, memory, motor skills, executive function, and behaviour at 3 to 5 years. We hypothesized that higher maternal BMI or GWG outside (i.e., below or above) the recommended range would be associated with lower scores on child performance measures of intelligence, language, memory, motor skills, and executive function, as well as poorer ratings on parent reports of executive function and behaviour in children. We also hypothesized that associations between maternal pre-pregnancy BMI and child intelligence, language, memory, motor skills, executive function, and behavioural outcomes would vary by GWG category.

## 2. Materials and Methods

### 2.1. Study Design and Participants

Pregnant women from Calgary and Edmonton, Canada (N = 2189) were recruited into The Alberta Pregnancy Outcomes and Nutrition (APrON) study between January 2009 and December 2012 [24]. Women who communicated in English were <27 weeks’ gestation and ≥16 years and whose children completed a neuropsychological assessment at 3 to 5 years participated (n = 379; 48.3% female; M age = 4.3, SD = 0.5). Children diagnosed with genetic or neurological diseases were excluded. The Conjoint Health Research Ethics Board at the University of Calgary (Ethics ID: REB14-1702) and the Health Research Ethics Board at the University of Alberta (Study ID: Pro00002954) approved the study. Women provide informed consent at the time of recruitment. Mothers provided informed consent for their child’s participation in the neuropsychological assessments.

### 2.2. Maternal Pre-Pregnancy Body Mass Index (BMI)

To determine maternal pre-pregnancy BMI, we used self-reported pre-pregnancy weight in kilograms and maternal height in metres measured at the time of recruitment. Women reported their pre-pregnancy weight in the month prior to pregnancy. Maternal self-reports of pre-pregnancy weight are valid measures of actual pre-pregnancy weight [25]. Maternal pre-pregnancy BMI was used in the main analyses. Due to the uneven distribution in the sample (i.e., 2.9% underweight, 62.5% normal weight, 22.2% overweight, 12.4% obese), BMI category, based on the definitions set by the WHO (i.e., underweight < 18.5 kg/m^2^; normal weight: 18.5–24.9 kg/m^2^; overweight: 25–29.9 kg/m^2^; obese ≥ 30 kg/m^2^) [26] was used in supplementary analyses that investigated the relationships among maternal GWG class and child neuropsychological outcomes.

### 2.3. Maternal Gestational Weight Gain (GWG)

GWG was estimated by subtracting the pre-pregnancy weight reported by the participant from the highest weight recorded (i.e., from medical records; weight directly measured by healthcare professionals) during the third trimester prior to delivery. Recommendations for GWG during pregnancy differ based on women’s BMI: 12.5–18.0 kg for underweight, 11.5–16.0 kg for normal, 7.0–11.5 kg for overweight, and 5.0–9.0 kg for obese [27]. These guidelines were used to classify GWG as below, within, or above recommended levels.

### 2.4. Child Neuropsychological Assessments

Children participated in assessments administered by a trained psychometrist and mothers completed questionnaires.

Intelligence was assessed with the Wechsler Preschool and Primary Scale of Intelligence, Fourth Edition, Canadian (WPPSI-IV^CND^). The WPPSI-IV^CND^ is a measure of intelligence in children ages 2:6 (years: months) to 7:7 [28]. We used the Full Scale IQ (FSIQ) and scores on three IQ indexes (i.e., Verbal Comprehension (VCI), Visual Spatial (VSI), Working Memory (WMI)). The scores are age-standardized (M = 100, SD = 15); higher scores indicate better performance. The WPPSI-IV^CND^ FSIQ score is a valid measure of intelligence and demonstrates excellent reliability, and the reliability of each of the indexes is acceptable [29]. FSIQ was the primary outcome; however, the indexes were also examined.

The Phonological Processing and Speeded Naming subtests of the Developmental NEuroPSYchological Assessment-Second Edition (NEPSY-II) were used to assess language. They are standardized for children aged 3:0 to 16:11 years [30]. Scaled scores are standardized by age (M = 10, SD = 3). Higher scores indicate better performance. The language subtests display adequate reliability in young children [31].

Memory was assessed using the NEPSY-II subtests: Memory for Designs, Narrative Memory, and Sentence Repetition. Scaled scores are standardized by age (M = 10, SD = 3), with higher scores indicating better performance. These subtests display adequate reliability in young children [31].

The Movement Assessment Battery for Children, Second Edition (MABC-2) was used to assess motor skills, specifically manual dexterity, aiming and catching, and balance [32]. In this study, the standard score (M = 10; SD = 3) for the Total Test score and standard scores (M = 10; SD = 3) on each domain were used. Higher scores are indicative of better performance. The MABC-2 is a valid and reliable measure of children’s motor skills [32].

The executive function of inhibitory control was assessed with the Boy–Girl Stroop, Less is More, and NEPSY-II Statue tests. Boy–Girl Stroop was adapted from the Day/Night task [33]. For each correct response, the child received one point. Less is More is a reverse reward contingency task [34], which requires conflict inhibition; the number of correct selections was recorded. NEPSY-II Statue measures motor inhibition and has high reliability [31]. An age-adjusted scaled score is calculated (M = 10, SD = 3); higher scores indicate better performance.

Children’s working memory was assessed with the Spatial Span and the Self-Ordered Pointing (SOPT) tests. On the Spatial Span test [35], the maximum number of span lengths that were correctly reproduced was recorded. For the SOPT [36], we recorded number of errors; higher scores indicate poorer performance.

Executive functions were also assessed with the Behavior Rating Inventory of Executive Function-Preschool Version (BRIEF-P) parent report. The BRIEF-P is a standardized 63-item inventory. It is valid and reliable for children 2.0 to 5.11 years [37]. Parents responded on a 3-point Likert scale (never = 1, sometimes = 2, often = 3) as to whether their child displayed specific behaviors. *T* scores were calculated for the General Executive Composite (GEC) and three indexes (i.e., Inhibitory Self-Control (ISC), Flexibility (FLEX), Emergent Metacognition (EMC)) (M = 50, SD = 10). On the BRIEF-P, higher scores denote greater difficulties.

Child behaviour was assessed using the Behavior Assessment System for Children-Second Edition (BASC-2), a valid and reliable measure of children’s behaviour [38]. Parents are asked to indicate on a rating scale (*never = 0, sometime = 1, often = 2, almost always = 3*), the occurrence of 134 different behaviours [38]. We used the *T* scores for the Internalizing Problems and Externalizing Problems scales (M = 50, SD = 10); higher *T* scores indicate more behaviour problems.

### 2.5. Covariates

Covariates included pertinent socioeconomic factors (i.e., annual household income, maternal education), maternal factors (i.e., parity, birthplace, age), child sex, and infant birthweight. As very few women reported smoking, consuming alcohol, and/or recreational drug use during pregnancy (<2%), these variables were not included as covariates. Dichotomous variables were coded as follows: annual household income (i.e., <$70,000 CAD versus >$70,000 CAD), maternal education (i.e., undergraduate university degree or higher versus trade school/high school diploma/lower), parity (i.e., primiparous versus multiparous), and maternal birthplace (i.e., Canadian born, not Canadian born).

### 2.6. Statistical Analyses

R (version 4.3.0) was used to conduct all analyses. Robust multivariable regressions with Huber M-estimation (using *rlm* from the *MASS* package) [39] were used. Three main models were examined: model 1 investigated the associations between continuous BMI and child neuropsychological outcomes; model 2 examined relationships between GWG class and child neuropsychological outcomes; and model 3 investigated associations between continuous BMI, GWG class, BMI × GWG class (interactions), and child neuropsychological outcomes. We used a Wald-type statistic (using *f.robftest* from the *sfsmisc* package) to determine robust *p*-values [40]. Significant interactions were probed using pairwise contrasts of estimated slopes (i.e., simple slopes by each GWG class) and interaction plots (using *emtrends* and *emmip* from the *emmeans* package) [41]. False discovery rate (FDR) was controlled using the Benjamini–Hochberg method [42]. Adjusted *p*-values (i.e., *q*-values) were computed; *q* < 0.10 were considered significant. There were few missing data (<2% missingness); chained equations using predictive mean matching (using *pmm* from the *mice* package) were used to impute missing data [43]. Supplementary analyses examined differences in the relationships between maternal GWG class and child neurodevelopment in sub-groups stratified by maternal BMI categories. These analyses were considered exploratory and should be interpreted cautiously. All main and supplementary models were adjusted for annual household income, maternal education, parity, maternal birthplace, maternal age, child sex, and birthweight. Models that included unstandardized executive function tasks were also adjusted for child age at assessment.

## 3. Results

### 3.1. Participant Characteristics

Mean maternal BMI was 24.6 kg/m^2^ (SD = 5.1, range: 16.4–46.4). Women’s mean age at recruitment was 32.3 years (SD = 3.8) (Table 1). Most of the women were born in Canada (83.1%), had an undergraduate university degree or higher (77.3%), had an annual household income ≥$70,000 CAD (85%), were primiparous (53%), and gave birth vaginally (77.3%). Regarding GWG, two-thirds (66.8%) of women gained outside the recommended range; 18.2% (n = 69) gained below, one-third (33.2%, n = 126) within, and almost half (48%, n = 182) gained above. The means of the children’s scores on the standardized neuropsychological measures were within the normal range (Table S1).

### 3.2. Model 1: Relationship between Maternal Pre-Pregnancy BMI and Children’s Neurodevelopmental Outcomes

In covariate-adjusted models, each unit increase in maternal BMI was linked to a 0.48-point (95% CI: −0.75, −0.21) decrement in children’s WPPSI-IV^CND^ FSIQ, and a 0.35 to 0.47-point decrement (95% CIs: −0.75, −0.02) on the VCI, VSI, or WMI indexes of the WPPSI-IV^CND^ (Table 2). Higher BMI was associated with poorer performance on the Phonological Processing and Sentence Repetition subtests of the NEPSY-II (*B*’s = −0.09 to −0.08, 95% CIs: −0.16, −0.02) (Table 3). On the MABC-2, higher BMI was related to lower Total and Manual Dexterity scores (*B*’s = −0.08 to −0.11, 95% CIs: −0.18, −0.01) (Table 3). On the executive function tasks, poorer performance on the Boy–Girl Stroop and Less is More (*B*’s = −0.16 to −0.09, 95% CIs: −0.26, −0.01) was associated with higher BMI (Table 4). Higher BMI was also associated with more parent-reported difficulties on the BRIEF-P GEC, and the ISC and EMC indexes (*B*’s = 0.24 to 0.37, 95% CIs: 0.05, 0.68) (Table 3). Maternal BMI was not related to extremizing or internalizing problems on the BASC-2 (Table 3). All significant associations survived FDR correction at *q* < 0.10.

### 3.3. Model 2: Maternal GWG and Children’s Neurodevelopment

In covariate-adjusted models, GWG below recommendations (i.e., GWG below), compared to gaining within recommendations (i.e., GWG within), was associated with a 4.04-point decrement (95% CI: −7.89 to −0.11) on the WPPSI-IV^CND^ FSIQ (Table 1). On the NEPSY-II, maternal GWG above recommendations (i.e., GWG above), compared to GWG within, was associated with lower scores on the NEPSY-II Phonological Processing and Narrative Memory subtests (*B*’s *=* −0.93 to −0.79, 95% CIs: −1.64, −0.06) (Table 2 and Table 3). Maternal GWG below, compared to GWG within, was associated with better performance on the Spatial Span test (*B* = 0.27, 95% CI: 0.02 to 0.52) (Table 4). Maternal GWG class was not associated with children’s motor performance or parents’ reports of executive function or behaviour (Table 3 and Table 4). No associations survived FDR correction.

### 3.4. Model 3: Interactions between Maternal BMI and GWG on Children’s Neurodevelopment

The influence of covariates is presented (Tables S2–S4). Significant interactions were noted for two intelligence and three executive function measures (Table 2 and Table 4). Probing of interactions revealed that interaction effects were similar for WPPSI-IV^CND^ indices (Figure S1, Table 5). Simple slopes analysis revealed that for women whose GWG was within recommendations, there was a significant effect of maternal BMI on VSI and WMI scores (*B*’s = −0.93 to −0.92, 95% CI: −1.55, −0.33). Specifically, for women in the GWG within group, lower BMI was associated with higher WPPSI-IV^CND^ subscale scores, whereas higher BMI was associated with lower scores. There was no or limited effect at GWG below or above recommendations (Figure S1, Table 5). For the NEPSY-II Statue subtest, simple slopes analysis revealed a significant effect for GWG within recommendations (*B* = −0.15, 95% CI: −0.29 to −0.02), and a trend for GWG below (*B* = −0.13, 95% CI: −0.27 to 0.02) (Figure S2, Table 5); for women in these groups, lower BMI was associated with higher Statue scores, whereas higher BMI was associated with lower scores. There was no or limited effects at GWG above recommendations. For Less is More and BRIEF-P ISC, the interaction effects were similar; simple slopes analysis showed that when GWG was below recommendations, lower BMI was associated with more correct responses on Less is More (*B* = −0.31, 95% CI: −0.49 to −0.13) and lower (i.e., fewer difficulties) ISC scores (*B* = 0.72, 95% CI: 0.34 to 1.10), while higher BMI was associated with poorer inhibitory control on these measures (Figure S2, Table 5). There was no or limited effect at GWG within or above recommendations. No interactions survived FDR correction. Visual inspection of the graphs showed that regression lines often intersected when maternal BMI was ≥25 (Figures S1 and S2); suggesting the negative effects on children’s intelligence and executive function may become evident when maternal BMI is in the overweight to obese range.

### 3.5. Supplementary Analyses

One-way ANOVAs showed differences in intelligence, memory, motor skills, and executive function scores between sub-groups stratified by maternal BMI (Table S1). Generally, children born to women with overweight and obese BMIs had poorer neuropsychological outcomes compared to those with normal BMIs.

The stratified regression models revealed that in women with normal BMIs, GWG below and above recommendations may be associated with lower child intelligence and language scores (Table S5). For example, in this sub-group, GWG below (95% CI: −10.03 to −1.07) and above (95% CI: −8.47 to −0.79) were associated with 5.6- and 4.6-point decrements on the WPPSI-IV^CND^ FSIQ, respectively. Stratified regressions also indicated that GWG below may be associated with poorer performance on intelligence, memory, and executive function measures in children born to women with obese pre-pregnancy BMIs, as well as more motor difficulties, externalizing problems, and executive function difficulties in children born to women with overweight BMIs (Tables S5–S7).

## 4. Discussion

Consistent with our hypotheses, we found that as maternal pre-pregnancy BMI increased, children’s scores on the WPPSI-IV^CND^, the gold standard for assessing intelligence in preschool-age children, decreased. Specifically, each unit increase in maternal BMI was associated with a 0.48-point decrement in children’s FSIQ, meaning that a 1-point increase in maternal BMI (e.g., 24.9 to 25.9) was associated with a half IQ point loss, while a 10-point increase in BMI (e.g., 24.9 to 34.9) was associated with a five-point loss in FSIQ. Similar findings were noted for the other WPPSI-IV^CND^ indices. A five-point loss in FSIQ for a child who is functioning in the average range of intelligence may not be clinically significant or influence long-term academic, occupational, or economic outcomes; however, among children who are functioning below average, such a loss in IQ could be detrimental to long term outcomes. Further, at the population level, the loss of IQ points and its association with increasing maternal BMI could have long-term impacts on productivity and economic growth.

Higher maternal BMI was also associated with poorer performance on measures of phonological coding, verbal memory, motor skills (particularly manual dexterity), and the executive functions of inhibitory control, emotional control, working memory, and planning/organizing. These findings extend those of previous studies [10,15,18] and systematic reviews [8,11,19], which have typically focused on the influence of maternal pre-pregnancy weight on child intelligence. They suggest that higher pre-pregnancy BMI is associated with poorer outcomes across a broad range of neuropsychological domains in preschool-aged children. These neurodevelopmental difficulties, particularly poorer cognitive outcomes, may place children at increased risk for developing mental health and behaviour problems in adolescence. Large cohort studies from Canada and the United Kingdom have reported that better cognitive abilities in early childhood are associated with decreased internalizing and externalizing symptoms in adolescents [44,45]. As accumulating evidence indicates that children born to women with obese pre-pregnancy BMIs are at increased risk of developmental adversities [46], additional research is needed to understand the pathways linking maternal obesity with neurodevelopmental difficulties and subsequent risk for emotional and behavioural problems.

We also found associations between maternal GWG and children’s intelligence and executive functioning, although these findings did not survive correction for false discovery rate (FDR) and will need to be replicated in future research. Specifically, maternal GWG below recommendations was associated with a 4.04-point decrement in children’s FSIQ (i.e., 4 IQ point loss) and poorer performance on a test of working memory, the Spatial Span. In contrast, a recently published systematic review reported no association between GWG below recommendations and children’s IQ, and a positive trend association between GWG above recommendations and children’s IQ [19]. Pugh et al. [18] reported associations between excessive GWG (i.e., >1 SD above the mean) and poorer working memory performance. The findings of the present study also revealed that GWG above recommended levels was associated with lower phonological processing and narrative memory scores. However, Martínez-Hortelano et. al. [19] found no support for an association between GWG above recommendations and language-related skills. This lack of consistency in the GWG literature could be due to several different factors, including differences in participants’ socioeconomic status, race/ethnicity, and pre-pregnancy weight. In addition, differences in the neuropsychological measures used to assess outcomes and variability in the covariates included in studies could account for the mixed results. Of note is the fact that the associations reported between GWG and children’s neurodevelopment are relatively weak. This could be due to the influence of pre- and postnatal factors that dilute the effect of GWG on child neurodevelopment. Many of our neuropsychological findings for maternal BMI and GWG were with respect to cognitive outcomes. This may be due to the measures included in this study, which assessed multiple cognitive domains (i.e., intelligence, memory, executive function). Overall, these findings highlight the sensitivity of offspring cognitive development to prenatal environmental conditions, such as maternal obesity, and reinforce the importance of applying developmental origins of health and disease (DOHaD) and life course theories in the context of neurodevelopment [7].

Previous research has not examined whether maternal BMI interacts with GWG class to predict children’s neurodevelopment. We found that higher BMI was associated with lower WPPSI-IV^CND^ scores among women whose GWG was within recommendations for their pre-pregnancy weight. Further, for women whose GWG was within or below recommendations, higher BMI was associated with poorer outcomes on several measures of inhibitory control (i.e., motor inhibition on NEPSY-II Statue, conflict inhibition on Less is More, inhibition and emotional control on BRIEF-P ISC). However, as these findings did not survive FDR correction, they will need to be replicated in future studies and should be interpreted with caution. However, they do suggest that when women gain weight within or below recommendations during pregnancy, higher pre-pregnancy BMI may confer a risk for lower intelligence and inhibitory control in children. It is notable that these adverse effects on child outcomes were often observed for women with maternal BMI ≥ 25 (see Figures S1 and S2). It is possible that suboptimal intrauterine conditions associated with maternal obesity, such as chronic inflammation [9,47] or “over nourishment” [48], which have been linked to other risk factors for poor cognitive outcomes in children (e.g., preterm delivery, low birthweight), could account for the lack of interactions between BMI and GWG above recommendations, and child neurodevelopment. It is also possible that the neurodevelopmental effects of GWG may differ based on the maternal pre-pregnancy BMI category. Our exploratory analyses suggested that insufficient or excessive GWG may confer neurodevelopmental vulnerability in children born to women with normal BMIs, while insufficient GWG may be a neurodevelopmental risk factor when women’s BMIs are in the overweight or obese ranges. Thus, clinicians may need to consider sub-group-specific recommendations, such as encouraging sufficient GWG to support healthy offspring neurodevelopment in women with overweight and obese pre-pregnancy BMIs. Future research that considers child-related factors such as low birthweight and postnatal variables such as the home environment and parental pre- and postnatal mental health are needed to untangle the complex relationships among pre-pregnancy BMI, GWG and children’s neurodevelopmental outcomes [49].

Maternal pre-pregnancy BMI is a composite of maternal genetic, sociodemographic, environmental, and lifestyle factors (e.g., diet, exercise) that can influence offspring growth and brain development [5,47]. Relatedly, maternal GWG is also associated with genetics, nutritional status, socioeconomic status, and systematic inflammation [5,9]. Maternal pre-pregnancy BMI reflects fat mass, while GWG reflects maternal fat accumulation, fluid expansion, and growth of the fetus, placenta, and uterus. Both pre-pregnancy BMI and GWG may have fetal programming effects on brain development through similar pathways, such as low-grade systematic inflammation or over- or undernutrition [48]. For example, maternal immune activation may disrupt maternal–placental–fetal interactions and reduce neural progenitor cell populations within the fetal brain [7]. The placenta is thought to mediate the effects of hyperlipidemia, systemic inflammation, and oxidative stress that characterize the obese maternal environment on the fetus, with increasing research indicating the epigenetic changes in the placenta may exert developing programming effects [50]. Collectively, our findings suggest that interventions (e.g., dietary, lifestyle) for pregnant women may need to be targeted based on maternal pre-pregnancy BMI and GWG during pregnancy. To better understand how maternal BMI and GWG influence children’s neurodevelopment, we need further research examining the complex interactions among biological mechanisms, and sociodemographic, psychosocial, and environmental factors associated with maternal weight and how they affect fetal brain development [5,46].

This study has several strengths including our use of robust models, our large sample size, our inclusion of relevant covariates such as annual household income and maternal education, and our examination of child neuropsychological outcomes across multiple domains. Over- or underestimation of pre-pregnancy weight and variability in the time at which the highest weight prior to delivery was recorded could lead to GWG misclassification and contribute to the mixed findings reported in research examining GWG and child neurodevelopment [19]. In the present study, maternal pre-pregnancy weight was obtained via self-report at the first study visit. The reliability of self-reported weight can vary at the extremes of bodyweight and based on sociodemographic factors; however, research suggests that maternal self-reports are valid measures of actual pre-pregnancy weight [25,51]. We used the final gestational weight recorded for each participant by their healthcare provider in the final month prior to delivery. Typically, most women in APrON saw their healthcare provider weekly within the final month of their pregnancy; therefore, the final gestational weight that was used to calculate GWG would provide a relatively accurate measure of their GWG over pregnancy. In our sample, we had a very small sub-group of women with underweight pre-pregnancy BMIs (n = 11); therefore, we recommend further examination of the associations between pre-pregnancy BMI and GWG, and the neuropsychological outcomes of children of women who were underweight prior to pregnancy. The generalizability of this study’s findings may be limited, given our relatively homogenous low-risk, high socioeconomic status, highly educated sample, although, there were still high rates of obesity and excessive weight gain among our participants (i.e., 34.6% of women had overweight or obese BMIs and 49.1% had GWG above recommended levels). The use of a low-risk cohort could account for the lack of associations between maternal BMI, GWG, and some neuropsychological outcomes, such as verbal IQ. However, the fact that we found associations between maternal pre-pregnancy BMI and various domains of neurodevelopment in this low-risk sample, provides strong support for the relationship between pre-pregnancy BMI and child neurodevelopment. Future research is encouraged that examines these associations in higher risk cohorts. A potential confounder that was not measured in the present study was maternal IQ, which is a well-known predictor of children’s intellectual, language, and executive function outcomes [52]. It is notable that most women in our sample had an undergraduate university degree, which would suggest at least an average IQ. However, even in this highly educated sample, we did control for maternal education, which is one of the most significant predictors of children’s cognitive outcomes [52] and is related to IQ. It is also possible that other unmeasured confounders may have affected the present results. For example, no data was collected on the time that parents spent engaging in physical activity with their children. It is possible that this unmeasured variable may impact children’s motor development. Future research is encouraged that examines additional factors that may impact not only children’s motor development but other areas of neurodevelopment.

## 5. Conclusions

Maternal pre-pregnancy BMI was associated with children’s intelligence, language, memory, motor, and executive function outcomes at ages 3 to 5. No associations were found for internalizing and externalizing behaviours. These findings lend support to the need to optimize maternal health and weight prior to conception. Additionally, the intrauterine conditions associated with insufficient or excessive GWG may confer neurodevelopmental risk; however, whether and how maternal BMI and GWG interact to influence offspring neurodevelopment is unclear and requires additional investigation. Larger prospective studies that comprehensively examine maternal weight-related determinants of children’s neurodevelopment and the mechanisms by which prenatal and postnatal environmental exposures can impact children’s brain development are needed.

## Figures and Tables

**Table 1:** Participant characteristics (N = 379).

Characteristics	N (%)
**Maternal** Age in years, M (SD)	32.34 (3.76)
Pre-pregnancy BMI, M (SD)	24.58 (5.06)
Underweight ^a^	11 (2.90)
Normal weight ^a^	237 (62.54)
Overweight ^a^	84 (22.16)
Obese ^a^	47 (12.40)
Birthplace	
Canadian Born	315 (83.11)
Not Canadian Born	57 (16.89)
Education	
High school diploma/trade school	86 (22.69)
Undergraduate university degree or higher	293 (77.31)
Annual household income	
<$70,000 CAD	57 (15.04)
≥$70,000 CAD	322 (84.96)
Parity	
Primiparous	201 (53.03)
Multiparous	178 (46.97)
Delivery mode	
Vaginal	293 (77.31)
Caesarean section	86 (22.69)
GWG Class ^b^	
Below	69 (18.21)
Within	126 (33.24)
Above	186 (49.08)
Alcohol consumption during pregnancy	7 (1.8%)
Smoking during pregnancy	6 (1.6%)
Recreational drug use during pregnancy	1 (0.03%)
**Child**	
Female	183 (48.28)
Birth weight in grams, M (SD)	3365.25 (529.90)
Age in years at Assessment, M (SD)	4.27 (0.50)

Abbreviations: M = mean; SD = standard deviation; BMI = body mass index. ^a^ Maternal pre-pregnancy BMI based on the WHO category definitions. ^b^ Maternal weight gain during pregnancy based on IOM 2009 guidelines.

**Table 2:** Covariate-adjusted robust regressions examining the associations between maternal pre-pregnancy body mass index (BMI), gestational weight gain (GWG) class, interaction terms, and children’s intelligence and language scores (n = 379).

	WPPSI-IV^CND^ FSIQ ^a^ *B* (95% CI)	WPPSI-IV^CND^ VCI ^a^ *B* (95% CI)	WPPSI-IV^CND^ VSI ^a^ *B* (95% CI)	WPPSI-IV^CND^ WMI ^a^ *B* (95% CI)	NEPSY-II Phonological Processing ^b^ *B* (95% CI)	NEPSY-II Speeded Naming ^b^ *B* (95% CI)
**Model 1 ^c^**						
Maternal BMI	−0.48 **^‡^ (−0.75, −0.21)	−0.47 **^‡^ (−0.75, −0.18)	−0.35 *^‡^ (−0.67, −0.02)	−0.36 *^‡^ (−0.67, −0.05)	−0.09 **^‡^ (−0.16, −0.03)	−0.03 (−0.09, 0.04)
**Model 2 ^d^**						
GWG Below	−4.04 * (−7.89, −0.11)	−1.46 (−5.58, 2.65)	−2.61 (−7.18, 1.97)	−4.27 (−8.68, 0.14)	−0.70 (−1.65, 0.24)	−0.54 (−1.42, 0.34)
GWG Met	Reference	Reference	Reference	Reference	Reference	Reference
GWG Above	−2.67 (−5.71, 0.38)	−1.67 (−4.85, 1.51)	−0.90 (−4.44, 2.64)	−1.78 (−5.18, 1.63)	−0.79 * (−1.52, −0.06)	0.02 (−0.67, 0.70)
**Model 3 ^e^**						
Maternal BMI	−0.78 ** (−1.30, −0.26)	−0.26 (−0.81, 0.29)	−0.93 ** (−01.55, −0.31)	−0.92 ** (−1.50, −0.33)	−0.12 (−0.25, 0.004)	−0.10 (−0.22, 0.02)
GWG Below	−9.69 (−28.1, 8.68)	9.44 (−9.84, 28.70)	−17.90 (−39.70, 3.89)	−18.9 (−39.50, 1.73)	−1.32 (−5.80, 3.16)	−3.41 (−7.52, 0.70)
GWG Met	Reference	Reference	Reference	Reference	Reference	Reference
GWG Above	−15.7 (−31.4, 0.07)	2.52 (−14.00, 19.00)	−20.80 * (−39.50, −2.13)	−20.3 * (−37.90, −2.61)	−2.35 (−6.19, 1.49)	−1.86 (−5.38, 1.66)
Maternal BMI × GWG Below	0.25 (−0.50, 1.01)	−0.45 (−1.24, 0.35)	0.67 (−0.23, 1.57)	0.64 (−0.21, 1.49)	0.03 (−0.16, 0.21)	0.13 (−0.05, 0.29)
Maternal BMI × GWG Met	Reference	Reference	Reference	Reference	Reference	Reference
Maternal BMI × GWG Above	0.56 (−0.08, 1.20)	−0.15 (−0.82, 0.52)	0.86 * (0.10, 1.62)	0.80 * (0.09, 1.52)	0.07 (−0.09, 0.23)	0.08 (−0.06, 0.23)

Abbreviations: WPPSI-IV^CND^ = Wechsler Preschool and Primary Scale of Intelligence-Fourth Edition, Canadian FSIQ = Full-Scale IQ; VCI = Verbal Comprehension Index; VSI = Visual Spatial Index; WMI = Working Memory Index; NEPSY-II = Developmental NEuroPSYchological Assessment-Second Edition. ^a^ Standard score; M = 100, SD = 15, Range = 40–160; ^b^ Scaled scores; M = 10, SD = 3, Range = 45–155; ^c^ Model 1 examines the independent associations between maternal BMI and child intelligence and language outcomes. ^d^ Model 2 examines the independent associations between maternal GWG class (below, met, above) and child intelligence and language outcomes. ^e^ Model 3 examines the interactive associations (i.e., product terms) between maternal BMI and GWG and child intelligence and language outcomes. * *p* < 0.05; ** *p* < 0.01; ^‡^ *q* < 0.10.

**Table 3:** Covariate-adjusted robust regressions examining the associations between maternal pre-pregnancy body mass index (BMI), gestational weight gain (GWG) class, interaction terms, and children’s memory, motor skills, and behaviour (n = 379).

	NEPSY-II Memory for Design ^a^ *B* (95% CI)	NEPSY-II Narrative Memory ^a^ *B* (95% CI)	NEPSY-II Sentence Repetition ^a^ *B* (95% CI)	MABC-2 Total ^a^ *B* (95% CI)	MABC-2 Manual Dexterity ^a^ *B* (95% CI)	MABC-2 Aiming and Catching ^a^ *B* (95% CI)	MABC-2 Balance ^a^ *B* (95% CI)	BASC- 2 Externalizing Problems ^b^ *B* (95% CI)	BASC-2 Internalizing Problems ^b^ *B* (95% CI)
**Model 1 ^c^**									
Maternal BMI	−0.05 (−0.10, 0.005)	−0.06 (−0.12, 0.01)	−0.08 **^‡^ (−0.15, −0.02)	−0.08 *^‡^ (−0.14, −0.01)	−0.11 **^‡^ (−0.18, −0.05)	−0.03 (−0.10, 0.04)	−0.03 (−0.08, 0.03)	0.15 ^‡^ (−0.02, 0.31)	0.02 (−0.17, 0.20)
**Model 2 ^d^**									
GWG Below	−0.31 (−1.09, 0.47)	−0.66 (−1.58, 0.26)	−0.75 (−1.65, 0.16)	−0.52 (−1.39, 0.36)	−0.52 (−1.49, 0.46)	0.27 (−0.70, 1.23)	−0.45 (−1.23, 0.34)	0.13 (−2.22, 2.48)	−2.42 (−5.07, 0.22)
GWG Met	Reference	Reference	Reference	Reference	Reference	Reference	Reference	Reference	Reference
GWG Above	−0.13 (−0.73, 0.47)	−0.93 ** (−1.64, −0.22)	−0.36 (−1.06, 0.34)	−0.35 (−1.03, 0.32)	−0.38 (−1.13, 0.37)	0.20 (−0.54, 0.95)	−0.24 (−0.84, 0.37)	0.82 (−1.00, 2.64)	−0.10 (−2.15, 1.95)
**Model 3 ^e^**									
Maternal BMI	−0.06 (−0.16, 0.05)	−0.03 (−0.15, 0.10)	−0.06 (−0.19, 0.06)	−0.11 (−0.23, 0.004)	−0.12 (−0.25, 0.01)	−0.09 (−0.22, 0.04)	−0.08 (−0.18, 0.03)	0.11 (−0.20, 0.43)	0.09 (−0.27, 0.44)
GWG Below	−0.22 (−3.90, 3.47)	1.22 (−3.14, 5.59)	0.91 (−3.39, 5.21)	−0.13 (−4.26, 4.01)	0.06 (−4.52, 4.65)	−0.41 (−4.93, 4.12)	−0.66 (−4.42, 3.11)	−8.27 (−19.30, 2.74)	−3.86 (−16.40, 8.70)
GWG Met	Reference	Reference	Reference	Reference	Reference	Reference	Reference	Reference	Reference
GWG Above	−0.51 (−3.67, 2.65)	−1.15 (−4.89, 2.59)	−0.10 (−3.79, 3.58)	−2.42 (−5.96, 1.12)	−0.96 (−4.88, 2.97)	−2.74 (−6.61, 1.14)	−2.54 (−5.77, 0.69)	2.79 (−6.64, 12.20)	4.60 (−6.15, 15.40)
Maternal BMI × GWG Below	−0.001 (−0.15, 0.15)	−0.08 (−0.26, 0.10)	−0.07 (−0.25, 0.11)	−0.01 (−0.18, 0.16)	−0.02 (−0.21, 0.17)	0.03 (−0.15, 0.22)	0.01 (−0.15, 0.17)	0.36 (−0.09, 0.81)	0.06 (−0.46, 0.58)
Maternal BMI × GWG Met	Reference	Reference	Reference	Reference	Reference	Reference	Reference	Reference	Reference
Maternal BMI × GWG Above	0.02 (−0.11, 0.15)	0.01 (−0.14, 0.16)	−0.01 (−0.16, 0.14)	0.09 (−0.06, 0.23)	0.03 (−0.13, 0.19)	0.12 (−0.03, 0.28)	0.09 (−0.04, 0.23)	−0.08 (−0.47, 0.30)	−0.19 (−0.63, 0.25)

Abbreviations: NEPSY-II = Developmental NEuroPSYchological Assessment-Second Edition; MABC-2 = Movement Assessment Battery for Children-Second Edition; BASC-2 = Behavior Assessment System for Children-Second Edition. ^a^ scaled scores; M = 10, SD = 3, Range = 45–155; ^b^ *T* scores; M = 50, SD = 10, Range = 20–100; scores less than or equal to 59 are considered in the normal range, 60–64 is considered mildly elevated, and scores greater than or equal to 65 are considered significantly elevated; ^c^ Model 1 examines the independent associations between maternal BMI and child memory, motor, and behavioural outcomes; ^d^ Model 2 examines the independent associations between maternal GWG class (below, met, above) and child memory, motor, and behavioural outcomes; ^e^ Model 3 examines the interactive associations (i.e., product terms) between maternal BMI and GWG and child memory, motor, and behavioural outcomes; * *p* < 0.05; ** *p* < 0.01; ^‡^ *q* < 0.10.

**Table 4:** Covariate-adjusted robust regressions examining the associations between maternal pre-pregnancy body mass index (BMI), gestational weight gain (GWG) class, interaction terms, and child performance on measures of executive function and parent reports of child executive function (n = 379).

	Boy/Girl Stroop ^a^ *B* (95% CI)	Less Is More ^a^ *B* (95% CI)	NEPSY-II Statue ^b^ *B* (95% CI)	SOPT ^c^ *B* (95% CI)	Spatial Span ^d^ *B* (95% CI)	BRIEF-P GEC ^e^ *B* (95% CI)	BRIEF-P ISC ^e^ *B* (95% CI)	BRIEF-P FLEX ^e^ *B* (95% CI)	BRIEF-P EMC ^e^ *B* (95% CI)
**Model 1 ^f^**									
Maternal BMI	−0.16 **^‡^ (−0.26, −0.07)	−0.09 *^‡^ (−0.17, −0.01)	−0.06 (−0.12, 0.01)	−0.01 (−0.04, 0.03)	−0.004 (−0.02, 0.01)	0.37 **^‡^ (0.16, 0.58)	0.24 *^‡^ (0.05, 0.43)	0.12 (−0.05, 0.29)	0.35 **^‡^ (0.23, 0.68)
**Model 2 ^g^**									
GWG Below	−0.80 (−2.14, −0.55)	−0.31 (−6.83, 4.51)	−0.17 (−1.15, 0.81)	−0.31 (−0.81, 0.20	0.27 * (0.02, 0.52)	0.87 (−2.15, 3.90)	0.50 (−2.18, 3.19)	0.66 (−1.79, 3.10)	0.88 (−2.36, 4.12)
GWG Met	Reference	Reference	Reference	Reference	Reference	Reference	Reference	Reference	Reference
GWG Above	−0.38 (−1.42, −0.66)	0.18 (−0.72, 1.08)	−0.05 (−0.70, 0.81)	−0.06 (−0.44, 0.33)	−0.02 (−0.22, 0.17)	1.51 (−0.83, 3.85)	1.78 ‡ (−0.30, 3.86)	0.16 (−1.73, 2.04)	1.57 (−0.94, 4.08)
**Model 3 ^h^**									
Maternal BMI	−0.19 * (−0.37, −0.02)	−0.01 (−0.19, 0.16)	−0.15 * (−0.29, −0.02)	0.03 (−0.04, 0.09)	0.002 (−0.03, 0.04)	0.31 (−0.10, 0.72)	0.14 (−0.22, 0.50)	0.16 (−0.17, 0.49)	0.36 (−0.08, 0.80)
GWG Below	−0.95 (−7.14, 5.25)	7.01 * (1.06, 13.00)	−0.75 (−5.43, 3.93)	−0.08 (−2.45, 2.29)	1.11 ‡ (−0.13, 2.34)	−9.85 (−24.30, 4.58)	−12.90 * (−25.50, −3.70)	−7.01 (−18.60, 4.62)	−5.96 (−21.30, 9.39)
GWG Met	Reference	Reference	Reference	Reference	Reference	Reference	Reference	Reference	Reference
GWG Above	−2.34 (−7.65, 2.96)	−0.39 (−5.49, 4.71)	−3.78 (7.79, 0.23)	1.34 (−0.69, 3.37)	−0.33 (−1.39, 0.72)	3.30 (−9.07,15.70)	3.19 (−7.56, 13.9)	4.28 (−5.68, 14.2)	0.24 (−12.90,13.4)
Maternal BMI × GWG Below	0.03 (−0.23, 0.28)	−0.30 * (−0.54, −0.05)	0.03 (−0.17, 0.22)	−0.01 (−0.11, 0.09)	−0.04 (−0.08, 0.02)	0.46 (−0.14, 1.05)	0.58 * (0.06, 1.09)	0.33 (−0.15, 0.81)	0.28 (−0.36, 0.91)
Maternal BMI × GWG Met	Reference	Reference	Reference	Reference	Reference	Reference	Reference	Reference	Reference
Maternal BMI × GWG Above	0.09 (−0.12, 0.31)	0.02 (−0.19, 0.23)	0.16 * (0.001, 0.33)	−0.06 (−0.14, 0.03)	0.01 (−0.03, 0.05)	−0.09 (−0.60, 0.41)	−0.06 (−0.50, 0.37)	−0.17 (−0.58, 0.23)	0.02 (−0.51, 0.56)

Abbreviations: NEPSY-II = Developmental NEuroPSYchological Assessment-Second Edition; SOPT = Self-Ordered Pointing Task; BRIEF-P = Behavior Rating Inventory of Executive Function-Preschool Version; GEC = Global Executive Composite; ISC = Inhibitory Self-Control; FLEX = Flexibility; EMC = Emergent Metacognition. ^a^ total correct responses; ^b^ scaled scores; M = 10, SD = 3, Range = 45–155; ^c^ total errors; ^d^ maximum achieved; ^e^ *T* scores; M = 50, SD = 10, Range = 20–100; scores less than or equal to 59 are considered in the normal range, 60–64 is considered mildly elevated, and scores greater than or equal to 65 are considered significantly elevated; ^f^ Model 1 examines the independent associations between maternal BMI and child performance on measures of executive function and parent reports of child executive function; ^g^ Model 2 examines the independent associations between maternal GWG class (below, met, above) and child performance on measures of executive function and parent reports of child executive function; ^h^ Model 3 examines the interactive associations (i.e., product terms) between maternal BMI and GWG and child performance on measures of executive function and parent reports of child executive function; * *p* < 0.05; ** *p* < 0.01; ^‡^ *q* < 0.10.

**Table 5:** Simple slopes analyses of the significant interactions between maternal pre-pregnancy BMI and GWG on child neurodevelopment.

Interaction	*B* (SE)	95% CI
Maternal BMI × GWG Class for WPPSI-IV VSI		
GWG Below	−0.26 (0.34)	−0.92, 0.40
GWG Met	−0.93 * (0.32)	−1.55, −0.31
GWG Above	−0.07 (0.23)	−0.52, 0.39
Maternal BMI × GWG Class for WPPSI-IV WMI		
GWG Below	−0.28 (0.32)	−0.90, 0.35
GWG Met	−0.92 * (0.30)	−1.50, −0.33
GWG Above	−0.11 (0.22)	−0.54, 0.32
Maternal BMI × GWG Class for NEPSY-II Statue subtest		
GWG Below	−0.13 (0.07)	−0.27, 0.02
GWG Met	−0.15 * (0.07)	−0.29, −0.02
GWG Above	0.01 (0.05)	−0.09, 0.11
Maternal BMI × GWG Class for Less is More task		
GWG Below	−0.31 * (0.09)	−0.49, −0.13
GWG Met	−0.01 (0.09)	−0.19, 0.16
GWG Above	0.003 (0.06)	−0.12, 0.13
Maternal BMI × GWG Class for BRIEF-P ISC index		
GWG Below	0.72 * (0.19)	0.34, 1.01
GWG Met	0.14 (0.18)	−0.22, 0.50
GWG Above	0.08 (0.13)	−0.19, 0.34

Abbreviations: SE = standard error; WPPSI-IV = Wechsler Preschool and Primary Scale of Intelligence-Fourth Edition^CND^; VSI = Visual Spatial Index; WMI = Working Memory Index; NEPSY-II = Developmental NEuroPSYchological Assessment Second Edition; BRIEF-P = Behavior Rating Inventory of Executive Function-Preschool Version; ISC = Inhibitory Self-Control; * *p* < 0.05.

## Data Availability

Data are contained within the article and Supplementary Materials.

## Supplementary Materials

The following supporting information can be downloaded at: https://www.mdpi.com/article/10.3390/children10121849/s1, Power Analysis; Figure S1: Interaction graphs showing the adjusted associations between maternal pre-pregnancy body mass index (BMI) and children’s scores on the WPPSI-IV Visual Spatial Index (A) and on the Working Memory Index (B) by maternal gestational weight gain (GWG) class; Figure S2: Interaction graphs showing the adjusted associations between maternal pre-pregnancy body mass index (BMI) and children’s scores on the NEPSY-II Statue subtest (A), Less if More (B) and the BRIEF-P Inhibitory Self-Control Index (C) by maternal gestational weight gain (GWG) class; Table S1: Child scores on the neurodevelopmental assessments for the overall sample and sub-groups stratified by maternal pre-pregnancy body mass index (BMI); Table S2: Influence of covariates in models which investigated associations between continuous maternal pre-pregnancy body mass index (BMI), gestational weight gain (GWG), interactions, and children’s scores on measures of intelligence and language (n = 379); Table S3: Influence of covariates in models which investigated associations between continuous maternal pre-pregnancy body mass index (BMI), gestational weight gain (GWG), interactions, and children’s scores on measures of memory, motor skills, and behavior (n = 379); Table S4: Influence of covariates in models which investigated associations between continuous maternal pre-pregnancy body mass index (BMI), gestational weight gain (GWG), interactions, and children’s scores on measures of executive function (n = 379); Table S5: Sub-group analyses stratified by maternal pre-pregnancy body mass index (BMI) examining the associations between gestational weight gain (GWG) class and children’s scores on measures of intelligence and language; Table S6: Sub-group analyses stratified by maternal pre-pregnancy body mass index (BMI) examining the associations between gestational weight gain (GWG) class and children’s scores on measures of memory, motor skills, and behavior. Table S7: Sub-group analyses stratified by maternal pre-pregnancy body mass index (BMI) examining the associations between gestational weight gain (GWG) class and child performance and parent report (BRIEF-P) measures of executive function.
