# Supplementary material for: Maternal Pre-Pregnancy BMI and Gestational Weight Gain Are Associated with Preschool Children’s Neuropsychological Outcomes in the APrON Cohort
Source: Children (Basel). 2023 Nov 25;10(12):1849. doi: 10.3390/children10121849 (PMC10742277; doi:10.3390/children10121849)
Supplement: Supplementary file 1 [file children-10-01849-s001.zip › children-2706144-supplementary.pdf]

Supplemental Material: Maternal Pre-Pregnancy BMI and Gestational Weight Gain Are Associated with Preschool Children's Neuropsychological Outcomes in the APrON Cohort

Gillian England-Mason[1,2], Alida Anderson[3], Rhonda C. Bell[4], Fatheema B. Subhan[5] Catherine J. Field[4], Nicole Letourneau[1,2,6,7,8], Gerald F. Giesbrecht[1,2,8,9], Deborah Dewey[1,2,8,10], and the APrON Study Team[11,12*]

[1] Department of Pediatrics, Cumming School of Medicine, University of Calgary, Calgary, AB *T2N 1N4*, Canada

[2] Owerko Centre, Alberta Children's Hospital Research Institute, University of Calgary, Calgary, AB T2N 1N4, Canada

[3] O'Brien Centre for the Bachelor of Health Sciences, Cumming School of Medicine, University of Calgary, Calgary, AB T2N 1N4, Canada
[4] Department of Agricultural, Food & Nutritional Science, University of Alberta, Edmonton, AB T6G 2R3, Canada
[5] Department of Nutrition and Food Science, California State Polytechnic University, Pomona, CA 91768, USA
[6] Faculty of Nursing, University of Calgary, Calgary, AB T2N 1N4, Canada
[7] Department of Psychiatry, Cumming School of Medicine, University of Calgary, Calgary, AB T2N 1N4, Canada
[8] Department of Community Health Sciences, Cumming School of Medicine, University of Calgary, Calgary, AB T2N 1N4, Canada
[9] Department of Psychology, Faculty of Arts, University of Calgary, Calgary, AB,T2N 1N4, Canada
[10] Hotchkiss Brain Institute, University of Calgary, T2N 1N4, Calgary, AB, Canada
[11] University of Calgary, Calgary, AB T2N 1N4, Canada
[12] University of Alberta, Edmonton, AB T6G 2R3, Canada

*A complete list of APrON Study team members appears in the Acknowledgements.

CONTENTS

CONTENTS	Page
Power Analysis	3
Figure S1	4
Figure S2	5-6
Table S1	7-9
Table S2	10
Table S3	11-12
Table S4	13
Table S5	14-15
Table S6	16-17
Table S7	18-19

Power Analysis

A power analysis was conducted to determine if our sample size was sufficient to detect a significant interaction between maternal pre-pregnancy body mass index (BMI) and gestational weight gain (GWG) on children's neurodevelopment. We used G*Power 3.1.9.7; linear multiple regression: fixed model, R^2 increase, with a medium effect size of 0.15, α of 0.05, power of 0.95, 3 tested predictors (i.e., BMI, GWG, interaction term), and 8 possible covariates for a total of 11 predictors. This analysis indicated that a sample size of 178 was sufficient; the present sample of 379 maternal-child pairs was well-powered to detect significant effects.

Figure S1. Interaction graphs showing the adjusted associations between maternal pre-pregnancy body mass index (BMI) and children's scores on the WPPSI-IV Visual Spatial Index (A) and on the Working Memory Index (B) by maternal gestational weight gain (GWG) class.

A) **B)**

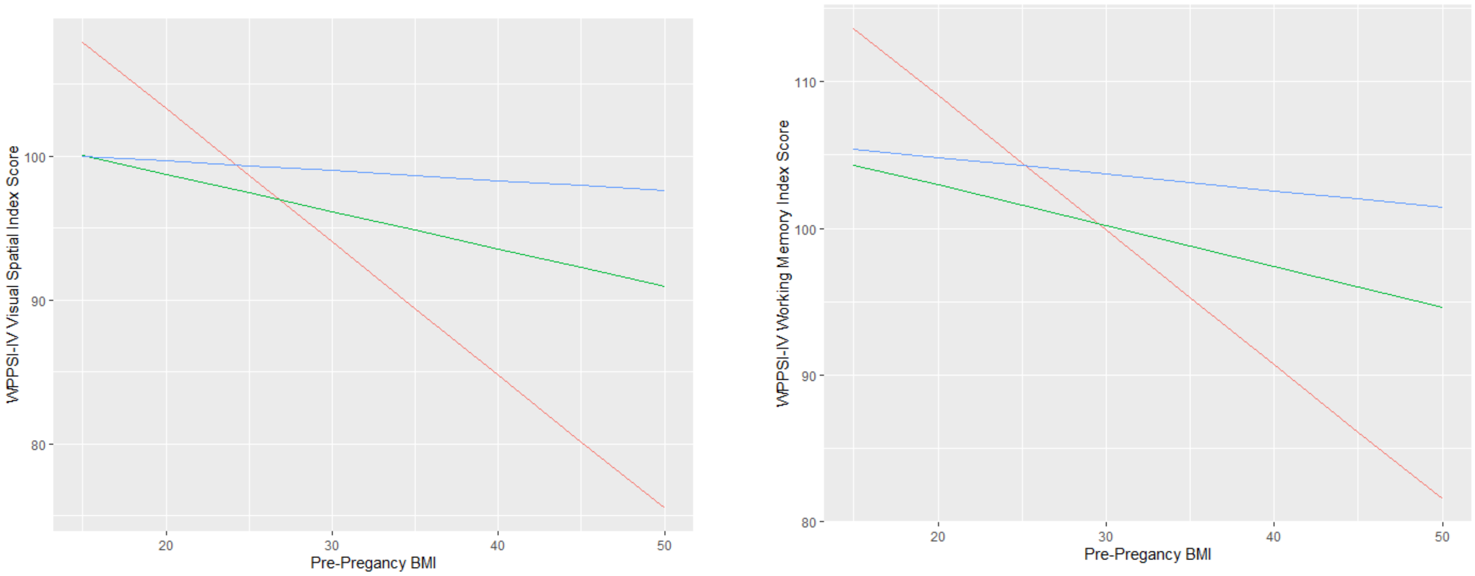

Note. Higher scores on the WPPSI-IV^{CND} indexes indicate better performance. Maternal GWG was classified as below (green line), within (red line), or above (blue line) the 2009 Institute of Medicine guidelines for recommended weight gain during pregnancy based on maternal pre-pregnancy BMI.

Figure S2. Interaction graphs showing the adjusted associations between maternal pre-pregnancy body mass index (BMI) and children's scores on the NEPSY-II Statue subtest (A), Less if More (B) and the BRIEF-P Inhibitory Self-Control Index (C) by maternal gestational weight gain (GWG) class.

A)

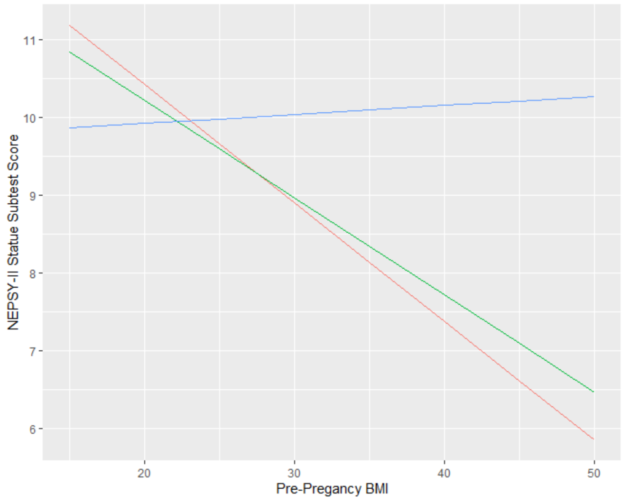

B)

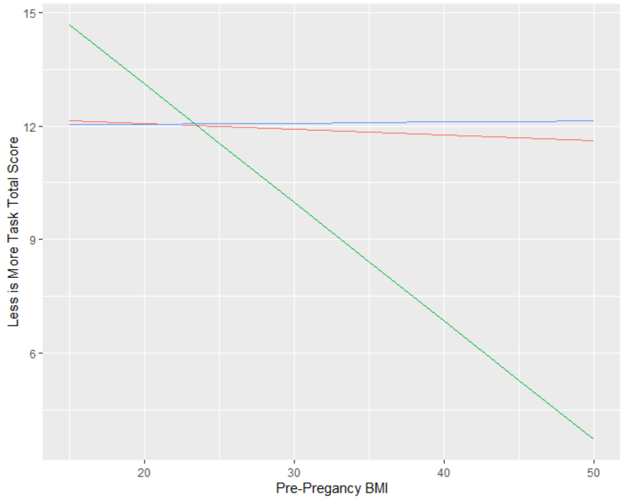

C)

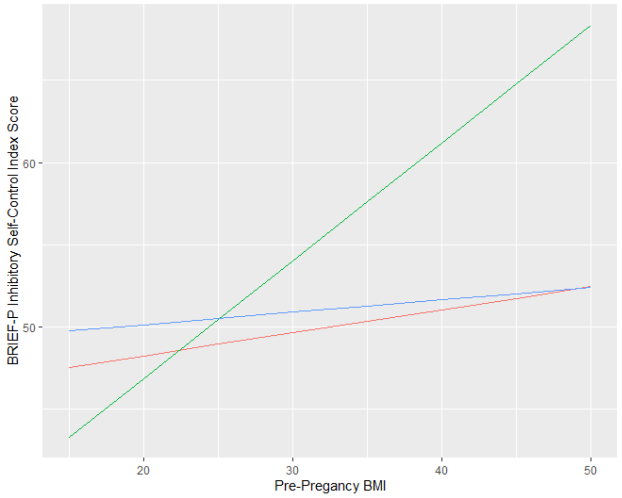

Note. A) Adjusted associations between maternal pre-pregnancy body mass index (BMI) and children's scores on the NEPSY-II Statue subtest by maternal gestational weight gain (GWG) class. B) Adjusted associations between maternal BMI and children's scores on the Less is More task based on maternal GWG class. C) Adjusted associations between maternal BMI and children's scores on the BRIEF-P Inhibitory Self-Control (ISC) index based on maternal GWG class. Higher scores on the Statue subtest and Less is More task indicate better performance; higher scores on the BRIEF-P ISC index indicate more parent-reported difficulties. Maternal GWG was classified as below (green line), within (red line), or above (blue line) the 2009 Institute of Medicine guidelines for recommended weight gain during pregnancy based on maternal pre-pregnancy BMI.

Table S1. Child scores on the neurodevelopmental assessments for the overall sample and sub-groups stratified by maternal pre-pregnancy body mass index (BMI).

	**Overall (N =379) M (SD)**	**Underweight (n = 11) M (SD)**	**Normal Weight (n = 237) M (SD)**	**Overweight (n = 84) M (SD)**	**Obese (n = 47) M (SD)**	***p*-value[a]**
**<u>Measures of Intelligence and Language</u>**						
WPPSI-IV[CND] FSIQ[b]	104.61 (13.63)	111.09 (11.40)	105.95 (13.22)	101.64 (13.53)	101.64 (15.08)	0.01*
WPPSI-IV[CND] VCI[b]	106.58 (13.79)	107.36 (6.65)	107.68 (13.41)	105.45 (14.98)	102.85 (14.26)	0.14
WPPSI-IV[CND] VSI[b]	102.74 (14.54)	112.55 (14.04)	103.59 (14.34)	99.15 (14.74)	102.57 (14.06)	0.01*
WPPSI-IV[CND] WMI[b]	105.07 (15.17)	110.91 (11.07)	105.67 (15.60)	103.83 (13.62)	102.94 (16.23)	0.32
NEPSY-II Phonological Processing[c]	10.07 (3.17)	10.45 (3.05)	10.30 (3.11)	9.69 (3.33)	9.51 (3.18)	0.26
NEPSY-II Speeded Naming[c]	11.98 (2.90)	12.55 (3.24)	12.10 (2.75)	11.63 (3.11)	11.89 (3.22)	0.56
**<u>Measures of Memory, Motor Skills, and Behaviour</u>**						
NEPSY-II Memory for Design[c]	9.91 (2.87)	12.82 (4.53)	9.88 (2.79)	10.00 (3.01)	9.19 (2.06)	< 0.01*
NEPSY-II Narrative Memory[c]	10.07 (3.03)	9.82 (3.03)	10.21 (3.11)	9.77 (2.95)	9.95 (2.82)	0.69
NEPSY-II Sentence Repetition[c]	10.88 (3.25)	11.73 (3.23)	11.18 (3.01)	10.11 (3.83)	10.53 (3.11)	0.04*
MABC-2 Total[c]	9.44 (2.99)	10.18 (3.09)	9.57 (3.01)	9.35 (2.91)	8.74 (3.01)	0.29

MABC-2 Manual Dexterity[c]	8.68 (3.23)	9.00 (3.29)	8.96 (3.29)	8.68 (3.03)	7.26 (2.96)	0.01*
MABC-2 Aiming and Catching[c]	9.63 (3.14)	9.00 (3.61)	9.76 (3.12)	9.54 (2.78)	9.28 (3.74)	0.67
MABC-2 Balance[c]	10.48 (2.92)	12.91 (3.88)	10.43 (2.86)	10.19 (2.89)	10.64 (2.90)	0.03*
BASC-2 Externalizing Problems[d]	47.93 (8.18)	49.64 (6.67)	47.24 (7.89)	48.50 (8.47)	50.02 (9.11)	0.13
BASC-2 Internalizing Problems[d]	47.66 (9.10)	50.18 (10.52)	47.74 (8.99)	46.23 (8.21)	49.19 (10.57)	0.23
**<u>Measures of Executive Function</u>**						
Boy/Girl Stroop[e]	10.68 (4.75)	13.09 (4.11)	10.97 (4.66)	10.32 (4.75)	9.32 (5.07)	0.04*
Less is More[e]	11.79 (4.32)	13.27 (3.98)	11.80 (4.31)	11.74 (4.17)	11.53 (4.73)	0.70
NEPSY-II Statue[c]	10.10 (3.33)	11.09 (2.55)	10.16 (3.17)	9.98 (3.66)	9.79 (3.69)	0.66
Self-Ordered Pointing Task[f]	3.77 (1.72)	3.73 (1.62)	3.78 (1.66)	3.77 (1.88)	3.77 (1.77)	0.99
Spatial Span[g]	2.67 (1.09)	2.91 (1.45)	2.70 (1.08)	2.61 (1.15)	2.55 (0.93)	0.68
BRIEF-P GEC[d]	50.58 (9.95)	47.45 (8.55)	49.52 (9.47)	51.60 (10.47)	54.85 (10.50)	< 0.01*
BRIEF-P ISC[d]	50.31 (9.53)	48.09 (7.84)	49.52 (9.34)	51.17 (9.81)	53.26 (9.84)	0.06
BRIEF-P FLEX[d]	48.28 (8.79)	46.36 (7.59)	48.22 (9.00)	47.77 (8.38)	49.93 (8.72)	0.48
BRIEF-P EMC[d]	51.96 (10.63)	49.00 (9.11)	50.46 (9.72)	53.65 (11.77)	57.21 (11.37)	< 0.001*

Abbreviations: SD = standard deviation; WPPSI-IV^{CND} = Wechsler Preschool and Primary Scale of Intelligence- Fourth Edition, Canadian; FSIQ = Full-Scale IQ; VCI = Verbal Comprehension Index; VSI = Visual Spatial Index; WMI = Working Memory Index; NEPSY-II = Developmental NEuroPSYchological Assessment- Second Edition; MABC-2 = Movement Assessment Battery for Children- Second Edition; BASC-2 = Behavior Assessment System for Children- Second Edition; BRIEF-P = Behavior Rating Inventory of Executive Function- Preschool Version; GEC = Global Executive Composite; ISC = Inhibitory Self-Control; FLEX = Flexibility; EMC = Emergent Metacognition.

[a]*p*-values for *F* statistics from one-way analysis of variance (ANOVA) for differences between maternal pre-pregnancy BMI stratified groups on continuous neurodevelopmental scores

[b]Standard score; M = 100, SD = 15, Range = 40-160

[c]Scaled scores; M = 10, SD = 3, Range = 45-155

[d]*T* scores; M = 50, SD = 10, Range = 20-100; scores less than or equal to 59 are considered in the normal range, 60-64 is considered mildly elevated, and scores greater than or equal to 65 are considered significantly elevated
[e]total correct responses
[f]maximum achieved
[g]total errors
*$p < 0.05$

Table S2. Influence of covariates in models which investigated associations between continuous maternal pre-pregnancy body mass index (BMI), gestational weight gain (GWG), interactions and children's scores on measures of intelligence and language (N = 379).

	**WPPSI-IV[CND] FSIQ[a]**	**WPPSI-IV[CND] VCI[a]**	**WPPSI-IV[CND] VSI[a]**	**WPPSI-IV[CND] WMI[a]**	**NEPSY-II Phonological Processing[b]**	**NEPSY-II Speeded Naming[b]**
	***B* (95% CI)**	***B* (95% CI)**	***B* (95% CI)**	***B* (95% CI)**	***B* (95% CI)**	***B* (95% CI)**
Annual household income	-7.63 (-11.35, -3.93)*	-9.28 (-13.19, -5.38)*	-7.04 (-11.46, -2.62)*	-3.83 (-8.01, 0.35)	-0.89 (-1.79, 0.03)	-1.33 (-2.17, 0.50)*
Maternal education	-4.36 (-7.60, -1.13)*	-3.92 (-7.32 -0.52)*	-3.11 (-6.96, 0.73)	-0.12 (-3.76, 3.51)	-0.47 (-1.26, 0.32)	-0.57 (-1.29, 0.16)
Parity	-0.10 (-2.93, 2.72)	-1.48 (-4.45, 1.48)	-1.23 (-4.58, 2.13)	1.59 (-1.58, 4.76)	0.00 (-0.69, 0.69)	-0.48 (-1.11, 0.15)
Maternal birthplace	-0.56 (-4.20, 3.09)	-2.96 (-6.79, 0.86)	-0.62 (-4.95, 3.71)	0.29 (-3.81, 4.38)	-0.94 (-1.83, -0.05)*	0.11 (-0.71, 0.92)
Maternal age	-0.17 (-0.55, 0.20)	-0.13 (-0.51, 0.26)	-0.03 (-0.47, 0.41)	-0.16 (-0.57, 0.26)	0.01 (-0.08, 0.10)	0.03 (-0.06, 0.11)
Child sex	-2.45 (-5.14, 0.23)	-3.70 (-6.51, -0.89)*	-2.40 (-5.59, 0.78)	-3.01 (-6.02, 0.00)	-1.23 (-1.88, -0.57)*	-0.27 (-0.87, 0.33)
Birthweight	0.00 (0.00, 0.01)	0.01 (0.00, 0.01)*	0.00 (0.00, 0.00)	0.00 (0.00, 0.01)	0.01 (0.00, 0.01)*	0.00 (0.00, 0.00)

Abbreviations: WPPSI-IV[CND] = Wechsler Preschool and Primary Scale of Intelligence- Fourth Edition, Canadian; FSIQ = Full-Scale IQ; VCI = Verbal Comprehension Index; VSI = Visual Spatial Index; WMI = Working Memory Index; NEPSY-II = Developmental NEuroPSYchological Assessment- Second Edition.
[a]standard score; M = 100, SD = 15, Range = 40-160
[b]scaled scores; M = 10, SD = 3, Range = 45-155
*$p < 0.05$

Table S3. Influence of covariates in models which investigated associations between continuous maternal pre-pregnancy body mass index (BMI), gestational weight gain (GWG), interactions and children's scores on measures of memory, motor skills, and behavior (N = 379).

	**NEPSY-II Memory for Design[a]** ***B* (95% CI)**	**NEPSY-II Narrative Memory[a]** ***B* (95% CI)**	**NEPSY-II Sentence Repetition[a]** ***B* (95% CI)**	**MABC-2 Total[a]** ***B* (95% CI)**	**MABC-2 Manual Dexterity[a]** ***B* (95% CI)**	**MABC-2 Aiming and Catching[a]** ***B* (95% CI)**	**MABC-2 Balance[a]** ***B* (95% CI)**	**BASC-2 Externalizing Problems[b]** ***B* (95% CI)**	**BASC-2 Internalizing Problems[b]** ***B* (95% CI)**
Annual household income	-0.44 (-1.18, 0.31)	-0.73 (-1.61, 0.16)	-1.02 (-1.89, -0.15)*	-0.73 (-1.56, 0.11)	-0.95 (-1.88, -0.02)*	-0.13 (-1.05, 0.78)	-0.38 (-1.14, 0.39)	1.17 (-1.07, 3.40)	1.08 (-1.46, 3.63)
Maternal education	-0.11 (-0.76, 0.54)	0.14 (-0.63, 0.91)	-0.05 (-1.26, 0.26)	0.05 (-0.68, 0.78)	-0.27 (-1.08, 0.53)	-0.27 (-1.07, 0.52)	0.34 (-0.32, 1.01)	1.81 (-0.13, 3.76)	1.59 (-0.63, 3.80)
Parity	0.11 (-0.45, 0.68)	0.48 (-0.19, 1.15)	0.12 (-0.54, 0.78)	-0.20 (-0.84, 0.44)	0.33 (-0.38, 1.03)	-0.51 (-1.21, 0.19)	-0.46 (-1.04, 0.12)	0.40 (-1.29, 2.10)	-2.10 (-4.03, -0.17)*
Maternal birthplace	0.44 (-0.30, 1.17)	-0.43 (-1.29, 0.44)	-0.42 (-1.27, 0.43)	0.51 (-0.31, 1.33)	0.22 (-0.69, 1.13)	0.63 (-0.27, 1.53)	-0.19 (-0.93, 0.56)	-0.44 (-2.62, 1.75)	-0.72 (-3.21, 1.77)
Maternal age	-0.05 (-0.13, 0.02)	-0.03 (-0.12, 0.05)	-0.05 (-0.14, 0.03)	-0.04 (-0.13, 0.04)	-0.04 (-0.13, 0.06)	-0.03 (-0.12, 0.07)	0.01 (-0.07, 0.08)	-0.13 (-0.35, 0.09)	-0.10 (-0.35, 0.16)
Child sex	0.00 (-0.54, 0.54)	-0.18 (-0.82, 0.46)	-1.05 (-1.68, -0.42)*	-1.36 (-1.97, -0.76)*	-1.81 (-2.48, -1.14)*	-0.40 (-1.06, 0.26)	-0.73 (-1.28, -0.18)*	2.11 (0.51, 3.72)*	-1.33 (-3.16, 0.51)
Birthweight	0.00 (0.00, 0.00)	0.00 (0.00, 0.00)	0.00 (0.00, 0.00)	0.00 (0.00, 0.00)	0.00 (0.00, 0.00)	0.00 (0.00, 0.00)	0.00 (0.00, 0.00)	0.00 (0.00, 0.00)	0.00 (0.00, 0.00)

Abbreviations: NEPSY-II = Developmental NEuroPSYchological Assessment- Second Edition; MABC-2 = Movement Assessment Battery for Children- Second Edition; BASC-2 = Behavior Assessment System for Children- Second Edition.
[a] scaled scores; M = 10, SD = 3, Range = 45-155

[b]*T* scores; M = 50, SD = 10, Range = 20-100; scores less than or equal to 59 are considered in the normal range, 60-64 is considered mildly elevated, and scores greater than or equal to 65 are considered significantly elevated
*$p < 0.05$

Table S4. Influence of covariates in models which investigated associations between continuous maternal pre-pregnancy body mass index (BMI), gestational weight gain (GWG), interactions and children's scores on measures of executive function (N = 379).

	Boy/Girl Stroop[a]	Less is More[a]	NEPSY-II Statue[b]	Self-Ordered Pointing Task[c]	Spatial Span[d]	BRIEF-P GEC[e]	BRIEF-P ISC[e]	BRIEF-P FLEX[e]	BRIEF-P EMC[e]
	***B* (95% CI)**	***B* (95% CI)**	***B* (95% CI)**	***B* (95% CI)**	***B* (95% CI)**	***B* (95% CI)**	***B* (95% CI)**	***B* (95% CI)**	***B* (95% CI)**
Annual household income	-0.64 (-1.93, 0.65)	-0.32 (-1.42, 0.79)	-0.99 (-1.94, -0.04) *	0.27 (-0.21, 0.75)	-0.25 (-0.49, 0.00)	1.85 (-1.08, 4.77)	0.54 (-2.01, 3.08)	0.65 (-1.71, 3.00)	2.59 (-0.52, 5.70)
Maternal education	0.44 (-0.69, 1.56)	0.11 (-0.85, 1.07)	0.05 (-0.78, 0.87)	0.01 (-0.41, 0.43)	-0.07 (-0.28, 0.14)	1.34 (-1.21, 3.88)	0.94 (-1.28, 3.15)	0.55 (-1.50, 2.60)	1.64 (-1.06, 4.35)
Parity	0.06 (-0.92, 1.05)	0.87 (0.03, 1.71) *	0.53 (-0.19, 1.25)	-0.01 (-0.38, 0.35)	0.10 (-0.08, 0.28)	-2.35 (-4.57, -0.13)*	-1.56, (-3.49, 0.37)	-2.56 (-4.35, -0.77)*	-2.34 (-4.70, 0.02)
Maternal birthplace	0.53 (-0.74, 1.80)	-0.09 (-1.17, 1.00)	-0.21 (-1.14, 0.72)	0.11 (-0.36, 0.58)	0.01 (-0.23, 0.24)	-0.29 (-3.16, 2.57)	-0.59 (-3.08, 1.90)	-1.11 (-3.41, 1.20)	0.53 (-2.52, 3.58)
Maternal age	-0.01 (-0.14, 0.12)	-0.01 (-0.12, 0.10)	-0.05 (-0.14, 0.05)	0.02 (-0.03, 0.07)	-0.02 (-0.05, 0.00)	-0.03 (-0.32, 0.26)	-0.16 (-0.41, 0.09)	-0.06 (-0.30, 0.17)	0.09 (-0.22, 0.40)
Child sex	-1.05 (-1.98, -0.12)	-0.38 (-1.18, 0.41)	-1.35 (-2.03, -0.67)*	-0.02 (-0.37, 0.032)	-0.13 (-0.30, 0.05)	1.36 (-0.74, 3.47)	1.86 (0.03, 3.69)*	2.41 (0.71, 4.10)*	0.40 (-1.85, 2.64)
Birthweight	0.00 (0.00, 0.00)	0.00 (0.00, 0.00)	0.00 (0.00, 0.00)	0.00 (0.00, 0.00)	0.00 (0.00, 0.00)	0.00 (0.00, 0.00)	0.01 (0.00, 0.01)*	0.00 (0.00, 0.00)	0.00 (0.00, 0.00)
Child age	2.52 (1.59, 3.45) *	3.42 (2.62, 4.21) *	-	-0.01 (-0.35, 0.34)	0.35 (0.18, 0.52) *	-	-	-	-

Abbreviations: NEPSY-II = Developmental NEuroPSYchological Assessment- Second Edition; BRIEF-P = Behavior Rating Inventory of Executive Function- Preschool Version; GEC = Global Executive Composite; ISC = Inhibitory Self-Control; FLEX = Flexibility; EMC = Emergent Metacognition.

[a]total correct responses

[b]scaled scores; M = 10, SD = 3, Range = 45-155

[c]total errors

[d]maximum achieved

[e]*T* scores; M = 50, SD = 10, Range = 20-100; scores less than or equal to 59 are considered in the normal range, 60-64 is considered mildly elevated, and scores greater than or equal to 65 are considered significantly elevated

Table S5. Sub-group analyses stratified by maternal pre-pregnancy body mass index (BMI) examining the associations between gestational weight gain (GWG) class and children's scores on measures of intelligence and language.

	**WPPSI-IV[CND] FSIQ[a]** *B* **(95% CI)**	**WPPSI-IV[CND] VCI[a]** *B* **(95% CI)**	**WPPSI-IV[CND] VSI[a]** *B* **(95% CI)**	**WPPSI-IV[CND] WMI[a]** *B* **(95% CI)**	**NEPSY-II Phonological Processing[b]** *B* **(95% CI)**	**NEPSY-II Speeded Naming[b]** *B* **(95% CI)**
**<u>Underweight (n = 11)</u>**						
GWG Below	56.78 (-418.37, 531.93)	-4.70 (-721.66, 712.27)	114.01(-1248.53, 1476.56)	20.84 (-888.87, 930.55)	42.30 (-177.10, 261.70)	8.75 (-273.44, 290.95)
GWG Met	Reference	Reference	Reference	Reference	Reference	Reference
GWG Above	-11.67 (-190.03, 166.70)	-3.66 (-272.80, 265.49)	-29.02 (-540.51, 482.47)	11.87 (-329.62, 353.37)	-16.23 (-177.10, 261.70)	9.62 (-96.31, 115.56)
**<u>Normal Weight (n = 237)</u>**						
GWG Below	-5.55 (-10.03, -1.07)*	-0.89 (-5.48, 3.71)	-5.17 (-10.17, -0.17)*	-6.16 (-11.58, -0.75)*	-1.08 (-2.15, -0.01)*	-1.14 (-2.10, -0.17)*
GWG Met	Reference	Reference	Reference	Reference	Reference	Reference
GWG Above	-4.63 (-8.47, -0.79)*	-1.58 (-5.51, 2.36)	-4.02 (-8.30, 0.27)*	-3.68 (-8.32, 0.96)	-1.09 (-2.00, -0.17)*	-0.46 (-1.29, 0.36)
**<u>Overweight (n = 84)</u>**						
GWG Below	7.94 (-4.77, 20.66)	6.00 (-8.74, 20.75)	5.15 (-10.61, 20.90)	2.03 (-12.18, 16.25)	1.35 (-2.33, 5.02)	2.36 (-0.79, 5.51)
GWG Met	Reference	Reference	Reference	Reference	Reference	Reference
GWG Above	0.97 (-5.53, 7.47)	-0.48 (-8.02, 7.05)	5.64 (-2.41, 13.69)	3.30 (-3.97, 10.56)	0.72 (-1.16, 2.60)	1.33 (-0.28, 2.94)

**Obese (n = 47)**						
GWG Below	-19.60 (-35.13, -4.06)*	-23.30 (-39.39, -7.20)*	-11.88 (-28.58, 4.82)	-1.71 (-20.24, 16.83)	-2.10 (-6.79, 2.59)	-1.35 (-4.65, 1.96)
GWG Met	Reference	Reference	Reference	Reference	Reference	Reference
GWG Above	-.18 (-10.48,10.84)	-6.29 (-17.33, 4.75)	1.65 (-9.80. 13.11)	5.90 (-6.81, 18.62)	-0.32 (-3.53, 2.90)	-0.55 (-2.82, 1.72)

Abbreviations: WPPSI-IV^{CND} = Wechsler Preschool and Primary Scale of Intelligence- Fourth Edition, Canadian; FSIQ = Full-Scale IQ; VCI = Verbal Comprehension Index; VSI = Visual Spatial Index; WMI = Working Memory Index; NEPSY-II = Developmental NEuroPSYchological Assessment- Second Edition.

[a] standard score; M = 100, SD = 15, Range = 40-160

[b] scaled scores; M = 10, SD = 3, Range = 45-155

*$p < 0.05$

Table S6. Sub-group analyses stratified by maternal pre-pregnancy body mass index (BMI) examining the associations between gestational weight gain (GWG) class and children's scores on measures of memory, motor skills, and behavior.

	NEPSY-II Memory for Design[a] *B* (95% CI)	NEPSY-II Narrative Memory[a] *B* (95% CI)	NEPSY-II Sentence Repetition[a] *B* (95% CI)	MABC-2 Total[a] *B* (95% CI)	MABC-2 Manual Dexterity[a] *B* (95% CI)	MABC-2 Aiming and Catching[a] *B* (95% CI)	MABC-2 Balance[a] *B* (95% CI)	BASC-2 Externalizing Problems[b] *B* (95% CI)	BASC-2 Internalizing Problems[b] *B* (95% CI)
**Underweight (n = 11)**									
GWG Below	0.17 (-595.67, 596.02)	-16.22 (-128.20, 95.77)	36.31 (-196.81, 269.42)	16.02 (-216.98, 249.03)	17.85 (-340.64, 376.33)	14.20 (-93.33, 121.72)	6.80 (-100.72, 114.33)	-27.94 (1049.64, 993.75)	34.08 (-978.37, 1046)
GWG Met	Reference	Reference	Reference	Reference	Reference	Reference	Reference	Reference	Reference
GWG Above	13.75 (-209.92, 237.43)	-2.13 (-44.17, 39.91)	-13.23 (-100.74, 74.28)	-1.85 (-89.32, 85.62)	-5.67 (-140.24, 128.90)	-1.03 (-41.39, 39.34)	6.03 (-34.34, 46.39)	3.00 (-380.53, 386.54)	-17.35 (397.41, 362.72)
Normal Weight (n = 237)									
GWG Below	-0.39 (-1.29, 0.51)	-0.62 (-1.72, 0.48)	-0.67 (-1.69, 0.34)	-0.70 (-1.75, 0.35)	-0.73 (-1.88, 0.42)	-0.11 (-1.20, 0.98)	-0.53 (-1.39, 0.33)	-0.39 (-2.89, 2.12)	-1.77 (-4.70, 1.15)
GWG Met	Reference	Reference	Reference	Reference	Reference	Reference	Reference	Reference	Reference
GWG Above	-0.23 (-1.00, 0.54)	-0.98 (-1.92, -0.04)*	-0.36 (-1.23, 0.51)	-0.66 (-1.56, 0.24)	-0.69 (-1.68, 0.30)	-0.08 (-1.02, 0.85)	-0.59 (-1.32, 0.15)	1.29 (-0.86, 3.44)	0.57 (-1.94, 3.07)
Overweight (n = 84)									
GWG Below	-0.04 (-3.10, 3.03)	0.57 (-2.43, 3.57)	-0.89 (-4.54, 2.76)	1.36 (-1.60, 4.32)	0.24 (-2.97, 3.45)	3.62 (0.56, 6.69)*	0.51 (-2.48, 3.49)	8.89 (1.70, 16.08)*	1.81 (-6.31, 9.94)
GWG Met	Reference	Reference	Reference	Reference	Reference	Reference	Reference	Reference	Reference
GWG Above	0.54 (-1.03, 2.10)	-0.42 (-1.95, 1.12)	1.01 (-0.85, 2.88)	0.72 (-0.80, 2.23)	0.92 (-0.72, 2.56)	1.27 (-0.29, 2.84)	0.36 (-1.16, 1.89)	-0.07 (-4.37, 2.98)	-0.73 (-4.88, 3.42)
Obese (n = 47)									
GWG Below	-2.65 (-5.29, -0.02)*	-3.26 (-6.46, -0.05)*	-5.01 (-8.74, -1.28)*	-1.75 (-5.06, 1.56)	-2.26 (-6.24, 1.72)	2.25 (-2.15, 6.66)	-2.78 (-6.22, 0.67)	9.38 (-2.92, 21.67)	-7.04 (-20.79, 6.71)
GWG Met	Reference	Reference	Reference	Reference	Reference	Reference	Reference	Reference	Reference
GWG Above	-0.97 (-2.77, 0.84)	-1.27 (-3.47, 0.93)	-2.26 (-4.82, 0.31)	0.26 (-2.01, 2.53)	-0.25 (-2.99, 2.48)	1.94 (-1.08, 4.96)	-0.11 (-2.47, 2.26)	3.12 (-5.32, 11.56)	1.73 (-7.71, 11.17)

Abbreviations: NEPSY-II = Developmental NEuroPSYchological Assessment- Second Edition; MABC-2 = Movement Assessment Battery for Children- Second Edition; BASC-2 = Behavior Assessment System for Children- Second Edition.

[a] scaled scores; M = 10, SD = 3, Range = 45-155

[b] *T* scores; M = 50, SD = 10, Range = 20-100; scores less than or equal to 59 are considered in the normal range, 60-64 is considered mildly elevated, and scores greater than or equal to 65 are considered significantly elevated

*$p < 0.05$

Table S7. Sub-group analyses stratified by maternal pre-pregnancy body mass index (BMI) examining the associations between gestational weight gain (GWG) class and child performance and parent report (BRIEF-P) measures of executive function.

	**Boy/Girl Stroop** [a]	**Less is More**[a]	**NEPSY-II Statue**[b]	**Self-Ordered Pointing Task**[c]	**Spatial Span**[d]	**BRIEF-P GEC**[e]	**BRIEF-P ISC**[e]	**BRIEF-P FLEX**[e]	**BRIEF-P EMC**[e]
	***B* (95% CI)**	***B* (95% CI)**	***B* (95% CI)**	***B* (95% CI)**	***B* (95% CI)**	***B* (95% CI)**	***B* (95% CI)**	***B* (95% CI)**	***B* (95% CI)**
**Underweight (n = 11)**									
GWG Below	44.34 (-336.67, 425.36)	4.42 (-578.04, 586.87)	2.38 (-163.36, 168.13)	-0.99 (-184.69, 182.71)	8.42 (-170.82, 187.67)	31.32 (-1128.91, 1191.55)	32.74 (-679.44, 744.92)	19.85 (-1198.71, 1238.42)	26.52 (-1026.19, 1079.23)
GWG Met	Reference	Reference	Reference	Reference	Reference	Reference	Reference	Reference	Reference
GWG Above	-9.96 (-152.98, 133.07)	4.07 (-214.58, 222.72)	6.86 (-55.36, 69.08)	1.06 (-67.90, 70.02)	0.16 (-67.12, 67.45)	-21.00 (-456.54, 414.54)	-13.90 (-281.25, 253.45)	-14.50 (-471.94, 442.94)	-25.03 (-420.20, 370.15)
**Normal Weight (n = 237)**									
GWG Below	-0.40 (-1.78, 0.99)	0.32 (-1.13, 1.76)	-0.19 (-1.27, 0.89)	-0.44 (-0.99, 0.11)	0.33 (0.04, 0.63)*	0.84 (-2.48, 4.15)	-0.24 (-3.32, 2.83)	0.58 (-2.25, 3.41)	0.99 (-2.47, 4.45)
GWG Met	Reference	Reference	Reference	Reference	Reference	Reference	Reference	Reference	Reference
GWG Above	-0.53 (-1.72, 0.66)	-0.53 (-1.76, 0.71)	-0.43 (-1.36, 0.49)	0.01 (-0.47, 0.48)	-0.16 (-0.41, 0.10)	1.45 (-1.38, 4.29)	1.52 (-1.12, 4.15)	0.83 (-1.59, 3.26)	1.55 (-1.42, 4.51)
**Overweight (n = 84)**									
GWG Below	-2.47 (-7.30, 2.36)	-2.00 (-6.34, 2.33)	0.19 (-3.74, 4.12)	0.79 (-0.92, 2.50)	-0.56 (-1.48, 0.36)	10.66 (1.18, 20.14)*	10.17 (1.14, 18.94)*	5.55 (-1.71, 12.82)	6.20 (-5.25, 17.65)
GWG Met	Reference	Reference	Reference	Reference	Reference	Reference	Reference	Reference	Reference
GWG Above	-0.29 (-2.76, 2.17)	0.51 (-1.71, 2.72)	0.03 (-1.97, 2.04)	0.43 (-0.45, 1.30)	-0.16 (-0.63, 0.31)	-1.66 (-6.51, 3.18)	0.50 (-3.98, 4.98)	-2.52 (-6.23, 1.19)	-2.53 (-8.38, 3.31)
**Obese (n = 47)**									
GWG Below	1.98 (-4.70, 8.66)	-5.09 (-10.81, 0.63)	0.33 (-4.22, 4.89)	-0.89 (-3.30, 1.52)	0.15 (-0.81, 1.11)	14.46 (-1.35, 30.27)	14.53 (1.72, 27.34)*	1.01 (-12.09, 14.11)	14.96 (-2.37, 32.29)

GWG Met	Reference	Reference	Reference	Reference	Reference	Reference	Reference	Reference	Reference
GWG Above	3.14 (-1.44, 7.73)	0.17 (-3.76, 4.09)	2.98 (-0.15, 6.11)	-1.31 (-2.97, 0.34)	0.28 (-0.37, 0.94)	6.98 (-3.87, 17.83)	5.97 (-2.82, 14.76)	2.90 (-6.09, 11.89)	9.51 (-2.38, 21.40)

Abbreviations: NEPSY-II = Developmental NEuroPSYchological Assessment- Second Edition; BRIEF-P = Behavior Rating Inventory of Executive Function- Preschool Version; GEC = Global Executive Composite; ISC = Inhibitory Self-Control; FLEX = Flexibility; EMC = Emergent Metacognition.

[a] total correct responses

[b] scaled scores; M = 10, SD = 3, Range = 45-155

[c] total errors

[d] maximum achieved

[e] *T* scores; M = 50, SD = 10, Range = 20-100; scores less than or equal to 59 are considered in the normal range, 60-64 is considered mildly elevated, and scores greater than or equal to 65 are considered significantly elevated

*$p < 0.05$
